# Climate Resilience: It Is Time for a National Approach

**DOI:** 10.1089/hs.2021.0108

**Published:** 2021-12-17

**Authors:** Scott D. Deitchman, Thomas D. Kirsch, Paul S. Auerbach, Alice C. Hill

**Affiliations:** Scott D. Deitchman, MD, MPH, RADM, USPHS (ret), is a Senior Visiting Scholar and Adjunct Associate Professor, and Thomas D. Kirsch, MD, MPH, FACEP, is a Professor and Director; both at the National Center for Disaster Medicine and Public Health, Uniformed Services University of the Health Sciences, Bethesda, MD. Paul S. Auerbach, MD, MS, was the Redlich Family Professor Emeritus, Department of Emergency Medicine, Stanford University School of Medicine, Stanford, CA. Alice C. Hill, JD, is the David M. Rubenstein Senior Fellow for Energy and the Environment, Council on Foreign Relations, Washington, DC.

**Keywords:** Climate change, Disaster preparedness, Community resilience, National strategy/policy, Public health preparedness/response

Human-induced climate change is increasing the frequency and severity of climate-related hazardous events. To prevent these events from becoming disasters, communities must identify, assess, and reduce their vulnerabilities to these hazards. Currently available resources are not coordinated across agencies and often are not validated, leaving community planners with the burden of determining which tools will be most useful for their individual needs. In this commentary, we briefly review federal resources available to American communities to guide those assessments and plans and recommend a national climate resilience strategy and adaptation resource center to support community planners.

## Climate Threats

Communities must urgently respond to the now more immediate threats posed by climate change. The 7 warmest years recorded in the United States since 1894 have all occurred since 2012, with 2020 being the fifth warmest year on record. Records for both the number of named hurricanes in the North Atlantic and the number of named storms making continental landfall were broken in 2020.^1^ In 2020, the United States experienced 22 climate-related disasters, each entailing losses of over US$1 billion, another annual record.^2^ Global average sea levels have risen 7 to 8 inches since 1900 and continue rising.^3^ Considering global land and surface temperatures, 2020 was the second-warmest year in 141 years and only 0.04°F (0.02°C ) below the record high set in 2016.^4^

While reducing greenhouse gas emissions is essential, the slow response of thermal masses influencing the earth's climate means that even if greenhouse gas concentrations are stabilized at current levels, the world may experience additional warming of 1.1°F (0.6°C) to 8.6°F (4.8°C).^5,6^ In the United States, heavy precipitation events are expected to increase in frequency and intensity in some regions. From 1961 to 2019, average heat wave frequency in the United States has increased from 2 to more than 6 per year, and the heat wave season is now 47 days longer.^7^ A long-duration drought already has developed in the American West, with wildfire incidence projected to increase in western states and Alaska.^8^ Models indicate an increase in tropical cyclone intensity and frequency.^9^ Other health threats associated with climate change include changes in the ranges of insect disease vectors; deaths and injuries from more frequent extreme events including heat waves, floods, and storms; increasing food insecurity; and decreased access to safe drinking water.^10^

Reducing greenhouse gases alone will not suffice to protect against climate hazards. Societies should reduce the risk of climate-related disasters by reducing community vulnerabilities to those hazards.

## Disaster Risk Reduction, Climate Change, and Health

Disasters inevitably affect humans and human health.^11^ Disaster risk is “the potential loss of life, injury, or destroyed or damaged assets which could occur to a system, society or a community in a specific period of time, determined probabilistically as a function of hazard, exposure, vulnerability, and capacity.”^12^ Disaster risk reduction seeks to reduce existing disaster risk, prevent new risks, and manage residual risk. Analogous to disaster risk reduction, climate change adaptation seeks to reduce vulnerability to current or expected climate and climate effects, including extreme climate events.

Disasters caused by climate-related hazards affect health in 2 ways: direct impact of the event that causes illness, injury, mental health trauma, or loss of life; and indirect impact on population health through damage to critical infrastructure that disrupts normal living conditions, critical services, and economic systems. The high winds and storm surges associated with tropical cyclones, for example, can kill and injure people directly but also damage critical infrastructure such as structures (including healthcare facilities), housing, utilities, and transportation, which disrupt access to basic services such as food, clean water, safe shelter, and healthcare. Community measures to reduce vulnerability to climate hazards protect public health by preventing disruption of these critical resources.

Hurricane Maria provided a powerful example of how a single climate-worsened event can negatively impact health both directly and indirectly. The category 4 hurricane made landfall in Puerto Rico on September 20, 2017, compounding the damage caused by Hurricane Irma 2 weeks earlier. Maria caused an estimated US$96.3 billion in damages to housing and critical infrastructure including the electricity and water supply, telecommunications networks, and access to medical care.^13,14^ In early December 2017, the official death count in Puerto Rico stood at 64.^15^ The island government raised the official death toll to 2,975 on August 28, 2018, based on a commissioned study and a number of other excess deaths studies that estimated increased mortality resulting from long-term health impacts and damage to critical infrastructure.^16-19^

Vast differences in death counts were the result of first reports only reflecting deaths directly related to the storm (eg, drowning, falls, crashes). Indirect deaths accounted for thousands of others, mostly because of the loss of electricity and damage to the healthcare system that prevented people from using medical equipment, securing pharmaceuticals, and accessing healthcare services. Other studies have found long-term increases in population mortality following disasters and damage to critical infrastructure.^20,21^

Disaster risk reduction strategies should focus on both individual and community protection. Individual protection against a hurricane includes, among other things, educating the population about climate risks, household preparedness, and early evacuation planning. Community risk reduction focuses on interventions such as protecting critical infrastructure, zoning laws to reduce development in at-risk areas, ensuring evacuation capabilities, and rapid recovery plans. Because excess morbidity and mortality are common after disasters, community preparedness often saves the most lives.

## Disaster Risk Reduction and Climate Change Adaptation Resources

Because climate risks are localized, communities are at the core of risk reduction. Communities must identify their vulnerabilities, assess how to reduce them, and implement plans. In the United States, various federal agencies have created diverse tools to aid community planners. While community planners benefit from the availability of these tools, the utility is impeded by an apparent lack of coordination and guidance.

The US Climate Resilience Toolkit^22^ is an interagency website developed under the auspices of the US Global Change Research Program and includes almost 500 “tools” drawn from various government agencies, universities, and nongovernmental organizations (NGOs). It also includes contact information for government experts who support climate vulnerability assessment and adaptation planning. The wealth of tools makes the toolkit a valuable resource, but potential community users may find it overwhelming even when using the various selection filters available. For example, a community planner in the American Southeast wishing to identify and address vulnerabilities to water supplies is presented with 13 tools that variously address flooding risk, water quality, drainage, coastal flooding, and water system vulnerability. At least 2 tools address drinking water vulnerability, leaving it to the user to determine which might be more suitable for their needs. The tool descriptions on the website do not indicate whether or how the tool was validated.

Additional tools are found on the websites of various federal agencies. A few of these are summarized in Table 1.

**Table 1.** Representative Climate Resilience Planning Tools from US Government Agencies

Source	Title	Year	Topic	Resource Description	Number of Planning Steps	Multistakeholder Process?	Specifically Addresses Underserved/Inequities?	Validation Process Explicitly Described?
United States Global Change Research Program	*US Climate Resilience Toolkit* ^22^	2014, revised 2016	Climate resilience in diverse settings	Collection of almost 500 diverse tools	5	Yes, in various tools	Yes, in various tools	Unclear and probably varies by tool
US Department of Homeland Security/Federal Emergency Management Agency	*National Mitigation Framework* ^23^	2013, updated 2016	Disaster risk reduction, mitigation and response	Broad conceptual presentation, not a detailed toolkit	Describes 7 necessary core capabilities	Yes, mentions need for diverse stakeholders	No	No
US Department of Homeland Security/Federal Emergency Management Agency	*Hazard Mitigation Assistance Grants (HMA), Building Resilience Infrastructures and Communities (BRIC)* ^24-26^	2003, revisions since	Grants programs	Disaster risk reduction grants programs; BRIC includes technical assistance	NA	NA, not a planning process	NA, not a planning process	NA, not a planning process
US Department of Transportation/Federal Highway Administration	*Highways in the Coastal Environment; Highways in the River Environment*; and associated climate guidance^27-30^	2012 to 2020	Highway planning, including mandate to include climate change	Planning guides	NA	Yes, mandated by Executive Order	No	Describe validation processes for recommended climate models and caution when a particular tool has not been validated
US Department of Commerce/National Institute of Standards and Technology	*Community Resilience Planning Guide* ^31^	2014, updated 2019	Community resilience	Community planning guide with associated resources	6	Yes	Yes, describes need to include “vulnerable populations”	No
US Department of Housing and Urban Development	*Community Resilience Toolkit* ^32^	Undated	Community resilience	Lists of hazards presented with possible assessment and mitigation strategies	NA, does not describe stepwise process	Yes	Yes, describes considering needs of “vulnerable populations”	No
US Environmental Protection Agency	*Regional Resilience Toolkit* ^33^	2019	Community resilience	Community planning guide with associated resources	5	Yes	Yes, specifically calls for equity and including disadvantaged populations	No, but toolkit was revised after pilot usage
US Environmental Protection Agency	*Climate Change Adaptation Resource Center* (*ARC-X*)^34^	Undated	Climate vulnerability assessment and adaptation	Collection of tools from EPA and other agencies	NA, does not describe stepwise process	Yes, in various tools	Yes, in various tools	Unclear and probably varies by tool
US Department of Health and Human Services/Centers for Disease Control and Prevention	*Building Resilience Against Climate Effects (BRACE) framework* ^35^	Undated, ongoing revisions	Health resilience	Climate resilience planning guide for health departments	5	Not explicitly described	Yes, mainly focused on contributors to health risk (eg, income)	Not specified, but lessons learned are shared among stakeholders

Abbreviation: ARC-X, Climate Change Adaptation Resource Center; BRACE, Building Resilience Against Climate Effects; BRIC, Building Resilience Infrastructures and Communities; EPA, US Environmental Protection Agency; HMA, Hazard Mitigation Assistance Grants; NA, not applicable.

## Assessed Status of Climate Adaptation Resources

American communities can choose from a plethora of available tools to assess their vulnerabilities to climate-related hazards and plan activities to reduce those vulnerabilities. Unfortunately, these tools vary widely, even in the frameworks they use. They vary in scope from holistic community planning resources to very specific resources, such as highway tools from the Federal Highway Administration and health tools from the US Centers for Disease Control and Prevention (Table 1).

In our review of these resources, 2 notable characteristics stood out. First, we could not readily find evidence that tools were developed in the context of a multiagency or “whole of government” program. Instead, most tools appear to have been created independently by agencies, even if a tool was developed through a multistakeholder process. Although the tools sometimes refer to tools from other agencies, there is no apparent overall strategy behind their intended uses or audiences. For example, the *US Climate Resilience Toolkit*^22^ provides aggregate information from across the US federal government but does not provide collaboratively developed resources designed to address climate threats holistically, despite the crosscutting threat of climate change. Second, tools provided by other agencies do not routinely mention a validation process, although a Federal Highway Administration tool describes validation processes for recommended climate models and caution when a particular tool has not been validated.^27^ Some agencies describe how they revised their tools based on stakeholder and expert input.^36^ Comparing tools would be easier if they were evaluated against a standardized dataset. However, tool descriptions on agency web pages typically did not mention evaluations, which users rely usually read to determine whether to explore particular tools. Indeed, others have observed that no such standardized external validation data exist.^37^ Because different assessment tools can produce very different results even when using the same data inputs, the lack of validation poses a burden for those who must select the tools used in their communities.^38^ We could not find any objective, consistent evaluation criteria for community leaders to determine which tools are best for their purposes.

## Assessment and recommendations

Since taking office, the Biden administration has emphasized the urgency of addressing climate change. The Executive Order on Tackling the Climate Crisis at Home and Abroad, issued on January 27, 2021, describes the mandate of a newly created interagency National Climate Task Force to include “planning and implementation of key federal actions to […] increase resilience to the impacts of climate change.”^39^ The administration directed agencies to develop their own climate adaptation plans and requested a US$14 billion budget increase for climate change mitigation and resilience.^40^ This effort has not yet produced a coordinated approach to resilience, however. All 10 “key planks” of the Biden administration's Climate Innovation Working Group address greenhouse gas reduction, but none mention climate vulnerabilities or disaster risk reduction.^41^ The discrepant tools offered by diverse agencies further demonstrate that the United States has not yet developed a unifying national climate resilience program designed to assist local communities.

We therefore propose 2 essential steps to reduce US communities' vulnerabilities to climate and other hazards: (1) create a national strategy for climate adaptation and risk reduction, and (2) develop a curated national resource of community climate adaptation tools.

### National Strategy for Climate Adaptation and Risk Reduction

The United States needs a clear strategy for climate adaptation and risk reduction that includes a whole of government approach.^42^ A multistakeholder, multiagency approach is required to harmonize and coordinate the laudable but diverse and often disconnected efforts by various departments and agencies and their NGO partners. Climate change hazards cut across the responsibilities of multiple agencies and mitigating them across the country presents fiscal challenges beyond the capabilities of individual agencies.^43^

For example, a coastal community may prioritize maintaining continuity of operations at its hospital during hurricanes, which are increasing in both frequency and severity. This can involve multiple topics spanning diverse agencies. Mitigating the risk of flooding that might affect the hospital may involve protecting natural coastal barriers, which involves programs of the US Environmental Protection Agency, the Department of Commerce/National Oceanic and Atmospheric Administration, the Department of the Interior, and NGOs such as the Nature Conservancy. Planning to ensure delivery of critical resources draws on the Federal Highway Administration and other Department of Transportation programs. Confirming that hospital structures can withstand anticipated storm-related stresses draws on research supported by the National Institute for Standards and Technology and the National Oceanic and Atmospheric Administration, while private architectural firms design the hospitals and retrofits. The US Department of Health and Human Services/Centers for Medicare and Medicaid Services sets emergency preparedness requirements for participating hospitals. The utilities that serve the hospital variously follow regulations and guidelines set by the US Department of Energy (electricity), the US Environmental Protection Agency (drinking water), and the Federal Energy Regulatory Commission and the US Department of Transportation Pipeline and Hazardous Materials Safety Administration (natural gas). Coordination between these agencies and topics likely would reduce duplication and result in better harmonized actions. Considering the likely large number of hospitals located in at-risk coastal communities, a coordinated approach also may provide efficiencies by sharing experience-based best practices.

Although the Obama administration issued Presidential Policy Directive 21 on Critical Infrastructure Security and Resilience to promote a national unity of effort toward resilient critical infrastructure, this policy did not explicitly mention climate change and did not include housing as “critical infrastructure.”^44^ Such a policy can inform broader national coordination of climate adaptation and risk reduction, which includes but is broader than infrastructure resilience.

The White House coordinates the executive branch, and the first order of business for any White House effort focused on climate resilience should be the creation of a national strategy for climate adaptation and risk reduction. This whole of government effort could be led by the White House Office of Domestic Climate Policy or the National Climate Task Force, ideally in coordination with the National Security Council, which has responsibility for White House policy on disaster preparedness and management. The organizers also can draw on expertise from academia, the private sector, and NGOs. Like Presidential Policy Directive 21, this climate resilience task force may identify specific leadership roles for particular departments or agencies.

The national strategy would help steer collaboration between and among the federal, state, local, and tribal governments, as well as NGOs and the private sector, as they devise policies to adapt and build resilience to climate impacts. It should include strategic plans for streamlining the federal funding procurement process for those communities and sectors that have the highest vulnerabilities and the least capacity to prepare for and cope with climate impacts. One key priority should be to synthesize climate information to create widely available, standardized national and regional climate data for use in local resilience planning efforts. This accumulation and synthesis of climate data would also help to improve models and predictive tools.

The national strategy should also foster development of a set of resilience metrics so that decisionmakers can more accurately measure community resilience, prioritize projects, and evaluate progress toward achieving greater resilience. A crucial role of the national strategy for climate adaptation and risk reduction will be to ensure that the policies and planning undertaken by federal, state, local, and tribal communities, NGOs, and private sector partners adequately address the inequities in the capacity for different communities to adapt to climate impacts. It should guide resource distribution to target disadvantaged and underserved communities so they also reap the benefits of adaptation and risk mitigation efforts.

### A National Climate Change Preparedness Resource Center

The diversity of current hazard vulnerability assessment and risk reduction planning tools demonstrate the need for a single national climate change adaptation resource center to which community leaders can turn. The functions of such a center could include:

- *Creating a curated collection of optimal tools for community planners* – This would include expert evaluation of tools for consistency, utility, and validity. It might be necessary to develop a standardized dataset, perhaps representing 1 or more fictional model communities, to evaluate tools being considered for inclusion.
- *Collecting and making available user evaluations of tools* – Community reports of their use of specific tools would be included in assessing each tool. Community planners would have access to both the tools and other users' experience to help each community identify which tools are best suited to its situation. Tool developers would have access to a growing body of user evaluations to guide further revision and improvement of the tools. Users would also benefit from experience-based data on the effectiveness of various interventions proposed in the tools.
- *Providing expert assistance in tool selection* – Because the collection will be actively curated, the expert curators will become familiar with the collection's contents. When community planners seek tools appropriate for their needs, the curators will help them narrow down the choices.
- *Providing expert assistance in data selection and acquisition* – Hazard vulnerability assessments require accessing and using diverse data sets including meteorology, seismic activity, vegetation and forestry, and land use. Data on disaster-related losses also help community planners assess and compare risks. Curators can help planners identify and access data sets appropriate to their needs.
- *Providing expert assistance in evaluation and planning* – Large communities may have full-time planning commissions, but this is less true of smaller communities. Even large cities may lack experience in formal assessments of climate-related hazards and disaster risk reduction. The climate change preparedness resource should therefore include a cadre of experts, already experienced in the use of various assessment and planning tools, who would be available to assist communities in their hazard vulnerability assessments and disaster risk reduction planning. These experts might be part of the climate change preparedness resource or be associated with partner organizations who routinely support local activities in public health and other needs.
- *Ensuring consideration of climate justice and equity*: Climate hazards do not affect all people equally. Social, political, and economic factors, as well as individual health conditions, increase vulnerabilities to climate-related hazards.^45^ The climate resilience resource must intentionally include tools that promote inclusion of the most vulnerable stakeholders and equitable climate adaptation.

No climate change resilience program can impose standardized solutions upon diverse, individual communities. Rather, the proposed center will provide consistency in resources, not tactics. The center should be a resource to help communities develop and work through the process by which they identify their own greatest hazards, their own vulnerabilities, and their own strategies to reduce those vulnerabilities.

The proposed center ideally would consist of full-time internal staff, including researchers, technical consultants to communities, and administrative leadership and support, and an external advisory committee of subject experts on retainer. The external advisors would help the program maintain high standards by advising on resource content, program priorities, and transparency, as do other advisory committees to government-supported programs.^46^ Such a center would require a substantial commitment of resources, particularly if it will provide comprehensive support to multiple communities. As severe climate events become more frequent, demand for the center's resources likely will increase; scaling up the center's capacity to meet that demand will require additional funding. As specific programs and priorities for the proposed center are determined through the development of a national climate adaptation strategy, it will be possible to estimate the funding needed for a center to support that strategy.

## Conclusion

Although the new administration's increased national focus on climate change mitigation through controlling greenhouse gas emissions is appropriate, reducing disaster risk by reducing climate-related vulnerabilities still appears to be a primarily community-level activity. However, because the resources currently available to community planners frequently are uncoordinated and lack validation, they are difficult to adapt at a community level. A national climate resilience policy that fosters effective collaboration among governments and nongovernmental partners would complement climate risk mitigation efforts by helping prepare the nation for climate hazards that will likely persist for decades. A national resource that assists communities in hazard vulnerability assessment and risk reduction planning—reducing the burden on individual communities to identify, evaluate, and learn how to use assessment and planning tools—could increase the number of communities that undertake and successfully complete these activities to reduce climate-related disaster risk. A national climate resilience policy and a planning resource to help enact that policy are essential to preparing for climate hazards that will challenge the world for decades to come.
